# Ultrafiltration of Rapeseed Protein Concentrate: Effect of Pectinase Treatment on Membrane Fouling

**DOI:** 10.3390/foods13152423

**Published:** 2024-07-31

**Authors:** Simone Bleibach Alpiger, Chloé Solet, Tem Thi Dang, Milena Corredig

**Affiliations:** Department of Food Science, Aarhus University, Agro Food Park 48, 8200 Aarhus, Denmark; chloe.solet@agrosupdijon.fr (C.S.); temdang@food.au.dk (T.T.D.); mc@food.au.dk (M.C.)

**Keywords:** rapeseed protein, pectinase, reversible fouling, irreversible fouling

## Abstract

Membrane filtration technologies have shown great potential as a gentle and effective method for concentrating and fractionating proteins for food applications. However, the application of this technology to plant-derived protein streams is in its infancy. In this study, an aqueous rapeseed protein concentrate was obtained with wet milling, and its performance during ultrafiltration with two distinct molecular weight cut-offs (10 and 100 kDa) was tested. All rapeseed proteins were retained during filtration. The addition of pectinase during extraction prior to filtration caused important structural modifications to the extract, resulting in increased permeate fluxes, increased carbohydrate permeation and a reduction in irreversible fouling. Lager pore sizes led to more pronounced fouling. FTIR analysis of the spent membranes showed that proteins and lipids are causing irreversible fouling.

## 1. Introduction

Membrane filtration technology has shown great potential as a gentle processing technology to separate and isolate proteins from plant materials [1,2]. Membrane filtration can provide means for protein concentration [3] while transmitting undesired small molecular weight components through the membrane into the permeate [4]. In addition, it has been suggested as a means for protein fractionation [5,6]. Fetzer et al. (2019) compared ultrafiltration (UF)-derived rapeseed protein isolates and those obtained through a conventional isoelectric precipitation. The results showed that while both methods produced protein fractions with similar protein profiles, the solubility of the UF-isolated proteins was significantly better than the precipitated counterparts [2]. These fractions were obtained from defatted press cakes and not from whole seeds. Another study using lentil protein isolates showed a shift in their pH-dependent solubility in the UF concentrates compared to isoelectric precipitates, suggesting differences in their structure after precipitation [1]. Hence, the exploration of membrane filtration represents an opportunity which may result in the creation of isolates and concentrates with improved techno-functional benefits, such as improved emulsifying, foaming, and gelling properties [1,7,8]. 

In industrial filtration processes, cross-flow filtration is commonly applied, where the feed flows tangentially to the membrane surface, and the permeate transmits through the membrane pores. Most of the existing literature has focused on the membrane processing of plant protein streams using UF membranes with relatively small pore sizes, which are typically characterized by a molecular weight cut-off (MWCO) ranging from 3 to 80 kDa. The predominant materials used for these membranes have been polyethersulfone (PES) and polysulfone (PS). The proteins are retained in the retentate, while small molecular weight compounds, such as anti-nutritional compounds, sinapic acid, and oligosaccharides, can be transmitted into the permeate stream, producing protein isolates [9,10]. 

Ultrafiltration may also be applied to less refined protein streams, yielding modification in composition and partial fractionations. For example, Ntone et al. (2020) reported the development of a rapeseed protein concentrate using a combination of ultrafiltration and diafiltration. This process increased the protein purity from 39.5% in the original feed stream to 65.1% protein per solids (*w*/*w*) by utilizing an ultrafiltration membrane with a small MWCO (5 kDa). In this case, the resulting protein concentrate included both the globulin and albumin fractions of rapeseed, namely cruciferin and napin, as well as a small fraction of oleosome-associated proteins oleosin. The fractionation of specific proteins may be achieved by employing membranes with larger pore sizes. For example, Ntone et al. (2021) utilized a 100 kDa ultrafiltration membrane to separate napin, recovering it in the permeate fraction while retaining other rapeseed proteins in the retentate.

During the filtration process, not all components will be transmitted through the membrane at the same rate, leading to concentration at the membrane surface, which is a phenomenon known as concentration polarization (CP) [11]. Formation of the CP layer occurs at the early stages of filtration, causing a reduction in the permeate flux to a steady-state regime. The CP layer affects membrane selectivity by acting as a secondary barrier to the permeation of various compounds. Over time, the highly concentrated layer at the membrane surface may turn into a gel layer, contributing to increased resistance to flow, which is also known as fouling [11]. Although these processes have been widely studied, details on the formation of CP layers in plant-derived streams are not yet available.

Plant protein extracts are often characterized by complex compositions of protein and other biomacromolecules, such as soluble fibers, and with proteins of polydisperse sizes and compositions. Hence, plant protein extracts are often composed of colloidal particles which can interact with the membrane in different ways depending on their physical and chemical properties [12,13]. The CP layer may lead to the deposition of colloidal material on the membrane, forming a cake layer and changing the nominal pore size of the membrane. This can reduce the permeate flux and alter the membrane selectivity [12]. Factors such as particle size, shape, pH, ionic strength, and particle electric charges play a significant role in the fouling mechanism [12]. Furthermore, the presence of lipids in the feed stream is known to cause challenges with fouling behavior [14]. Determining fouling and foulant compositions is therefore essential. 

A promising approach involves combining enzymatic treatments with membrane filtration, offering numerous possibilities for optimizing production [15] and creating new functional protein fractions [16]. One approach involves integrating enzymes into the cleaning procedure [17,18], while others have shown a positive impact on permeate fluxes during filtration through the incorporation of carbohydrases [19,20]. In this work, we propose that an enzyme treatment before membrane filtration may offer numerous possibilities for optimizing production (e.g., reducing fouling, improving extraction yields and resource utilization) as well as create new functional protein fractions [16]. Advances in membrane filtration technology, combined with gentler fractionation methods like wet milling or enzymatic transformations, have the potential to enhance the quality and functionality of plant-derived protein ingredients. Therefore, the aim of this research was to examine the influence of introducing a commercial pectinase to a mildly refined rapeseed protein concentrate on membrane fouling behavior during UF filtration, utilizing two distinct pore sizes. 

## 2. Experimental Methods

### 2.1. Protein Extraction 

A protein concentrate was obtained through the wet milling of whole rapeseeds (8.0% moisture, 48.8% oil and 15.7% protein) as previously published [21]. The rapeseeds were donated by Scanola A/S (Aarhus, Denmark). In brief, seeds, initially cracked using a Thermomix TM6 (Vorwerk, Wuppertal, Germany) were dispersed in MilliQ water at a concentration of 40% (*w*/*w*) and milled using an Ultra-Turrax T-25 (IKA, Staufen, Germany) operating at 13,500 rpm for 2 min. The resulting suspension was diluted to 10% (*w*/*w*) solids and subjected to further milling, using the same conditions. The suspension was mixed for 4 h at room temperature using a magnetic stirrer (MIX 15 eco, 2 mag, Muenchen, Germany) set at 350 rpm, with or without pectinase (Pectinex Ultra SP-L, 3300 units/g, Novozymes A/S, Bagsvaerd, Denmark) added, at a ratio of 100 mg/g rapeseed. The slurry was then processed through a twin-screw press (Angel Juicer 7500, Angel, Naarden, The Netherlands). This process resulted in a press cake and a liquid extract, which was subjected to centrifugation at 3500× *g* for 30 min at 4 °C (Megafuge ST4 Plus, ThermoFisher, Waltham, MA, USA). Three phases were obtained after centrifugation: a lipid layer at the top, a soluble fraction (subnatant) representing the protein concentrate, and a precipitate at the bottom. The subnatant was further processed by membrane filtration.

### 2.2. Ultrafiltration

**Figure 1:**
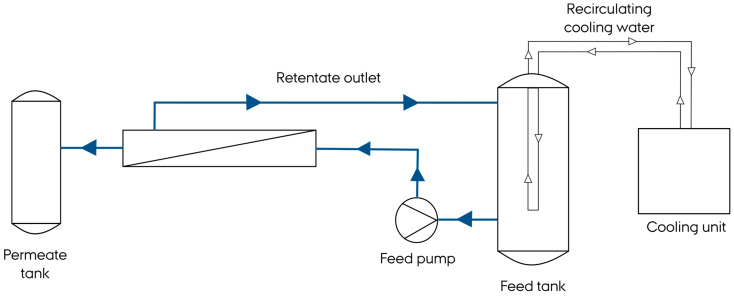
Schematic overview of filtration setup.

Ultrafiltration experiments were conducted using a cross-flow ultrafiltration unit, as illustrated in Figure 1 (CUBE 80VA, SIMA-tec, Schwalmtal, Germany), equipped with flat sheet PES membranes (Synder Filtration, Vacaville, CA, USA), 85 cm^2^, with two different molecular weight cut-off (MWCO) values, 10 kDa and 100 kDa. The temperature was maintained at 22 °C throughout filtration using a thermostat (Ecoline R306, LAUDA, Marlton, NJ, USA). For ultrafiltration with a 10 kDa membrane, the following conditions were applied: transmembrane pressure (TMP): 10 ± 0.2 bars and feed flow rate: 38 ± 1 L/h. In the case of the 100 kDa ultrafiltration membrane experiments, the operational conditions were as follows: TMP: 4 ± 0.2 bars and feed flow rate: 38 ± 1 L/h. In each trial, one liter of protein concentrate was used, and filtration experiments were carried out for 3 h. The permeate flux, J, was continuously recorded. 

The concentration factor was estimated based on permeate volume and calculated as follows:

(1) $$Volumetric\;concentration\;factor=\frac{V_{feed}}{V_{feed}-V_{permeate}}$$

where *V_feed_* is the volume of the initial feed and *V_permeate_* is the volume recovered in the permeate fraction. 

After the filtration experiment, the membranes were subjected to a cleaning process to assess irreversible fouling. This process was in accordance with previous literature [22,23] and involved a sequence comprising water rinsing (15 min), alkaline solution (NaOH, pH 11) recirculation (30 min), water rinsing (15 min), acid (HCl, pH 2) cleaning (30 min), and a final water rinse (15 min). The feed flow rate was maintained at 38 L/h with pressures set at 10 bar for 10 kDa UF and 4 bar for 100 kDa UF.

The fouling resistance (*R_t_*), which is the sum of reversible and irreversible fouling (*R_r_* and *R_ir_*), was calculated as the relative difference between flux before and after filtration as follows:

(2) $$R_{t}=R_{r}+R_{ir}=\frac{{J_{w1}-J}_{p}}{J_{w1}}$$

where *J_w_*_1_ is the initial permeate flux with water, before filtration, *J_p_* is the permeate flux at the end of the run, *J_w_*_2_ is the flux after cleaning, and *R_r_* and *R_ir_* are estimated as follows:

(3) $$R_{r}=\frac{J_{w2}-J_{p}}{J_{w1}}$$

(4) $$R_{ir}=\frac{J_{w1}-J_{w2}}{J_{w1}}$$

In addition, the flux recovery ratio (*FRR*) was calculated by the ratio between the permeate flux after cleaning and permeate flux before filtration:

(5) $$FRR=\frac{J_{w2}}{J_{w1}}$$

### 2.3. Compositional Analysis

The dry matter content of both seeds and liquid samples was determined gravimetrically by evaporating the moisture in an air oven at 105 °C until stable dry weight, according to AACC 44.15.02 (1999). Oil content was quantified through 4 M HCl hydrolysis followed by Soxhlet extraction with petroleum ether using a Hydrotherm (HT6, Gerhardt GmbH & Co. KG, Königswinter, Germany) and a Soxtherm (Gerhardt GmbH & Co. KG). The protein content of seeds and liquid samples was measured using the nitrogen combustion method (Dumas) with a Dumatherm N Pro (Thermo Scientific, Waltham, MA, USA), employing a conversion factor of 5.7 [2]. The total carbohydrate content was estimated using the phenol sulfuric acid method [24], using glucose as standard. To assess the composition of the protein in the retentates and permeates, SDS-PAGE was carried out under non-reducing conditions as previously described in the literature [21]. 

### 2.4. FTIR 

The composition of the fouling layer was analyzed by ATR-FTIR. The membranes, exposed to filtration and cleaning, were air-dried and three samples were taken from the top, middle, and bottom sections of the flat sheet. New membranes soaked in water for 24 h, air-dried and used as reference. The ATR-FTIR analysis was conducted using a Spectrum 3 Mid-IR Spectrometer by Perkin Elmer (Waltham, MA, USA) with a spectral resolution of 0.4 cm^−1^ for the 3028 cm^−1^ band in methane. A macro-ATR unit equipped with a thallium bromoiodide (KRS-5) was used for the attenuated total reflectance (ATR) experiments. To ensure consistent and close contact with the diamond, a sample press device was employed, facilitating the acquisition of a reliable signal. A uniform force of 10 bar was applied for all measurements, and the settings included a resolution of 4 cm^−1^ and 32 scans. Data were evaluated using MATLAB (version 9.14.0.2254940, R2023a). Preprocessing methods were applied to the spectra, including Savitzky–Golay noise reduction, multiplicative scattering correction, and second-order derivative. The analysis focused on the 4000–900 cm^−1^ spectral region. Secondary structure analysis of the protein present in the membranes was performed by band deconvolution of the amide I band (1700–1600 cm^−1^) using MATLAB (version 9.14.0.2254940, R2023a). A linear baseline correction was applied to the amide I data, which was followed by a second-order derivative to identify initial peak positions. The relative abundance of the secondary structure was calculated by Gaussian deconvolution of the amide I peak. 

### 2.5. Statistics 

Results are presented as mean values ± standard deviations of two independent experiments. Statistical analysis for the secondary structure components was carried out with SAS software (version 9.4, SAS^®^ Institute Inc., Cary, NY, USA). One-way ANOVA followed by Tukey’s test was applied to evaluate significant variations among the means (*p*-value < 0.05). 

## 3. Results and Discussion 

### 3.1. Rejection and Permeation of Components during Filtration

Rapeseed protein concentrates were filtered with two different PES membranes of 10 and 100 kDa MWCO. The composition of the feed is summarized in Table 1. The addition of pectinase during extraction increased the dry matter concentration and the ratio of carbohydrates to proteins in the feed. Consequently, the extract subjected to pectinase treatment was diluted to achieve a comparable solids concentration to the control, ensuring the best comparability during filtration (Table 1). Retentate and permeate streams were collected continuously for the whole duration (3 h) of the filtration experiment, which reached a concentration factor between 1.2 and 1.4, which was based on the permeate volume. The lowest concentration factor was observed in the control feed using the 10 kDa membrane, while higher levels were detected for the extracts treated with pectinase. In addition, the concentration ratio of dry matter was calculated with values close to 1 indicating a loss of dry matter in the retentate during filtration. Conversely, dry matter was found to be recovered in the permeate streams, as shown in Table 1. Increasing the pore size from 10 to 100 kDa enhanced the permeation of dry matter in both concentrates. The control retentate showed a more pronounced effect with the amount of permeated solids rising from approximately 2% to 8% of the initial dry matter. In all cases, about 25% of the solids in the initial feed was lost during filtration, implying that solids were retained in the membrane pores or in the cake layer.

The most notable differences in the retentates were related to the carbohydrate concentration in the feed after filtration. When subjected to ultrafiltration with a 100 kDa membrane, the rapeseed protein concentrate experienced a decrease in carbohydrate concentration of about 24% (Table 1). The same reduction occurred in the presence of pectinase, and in this case, the decrease was observed after filtration with both 10 kDa and 100 kDa UF membranes, leading to reductions of 16% and 27%, respectively. This suggests that the enzymatic-assisted extraction led to a reduction in the molecular size of the soluble carbohydrates, allowing for more dry matter reduction, in particular when employing the 10 kDa membranes. Pectinex, the pectinase utilized in this study, is a broad-spectrum enzyme with a primary specificity for the galacturonic acid backbone of pectic polysaccharides [25], resulting in the fragmentation of these polysaccharides. 

**Figure 2:**
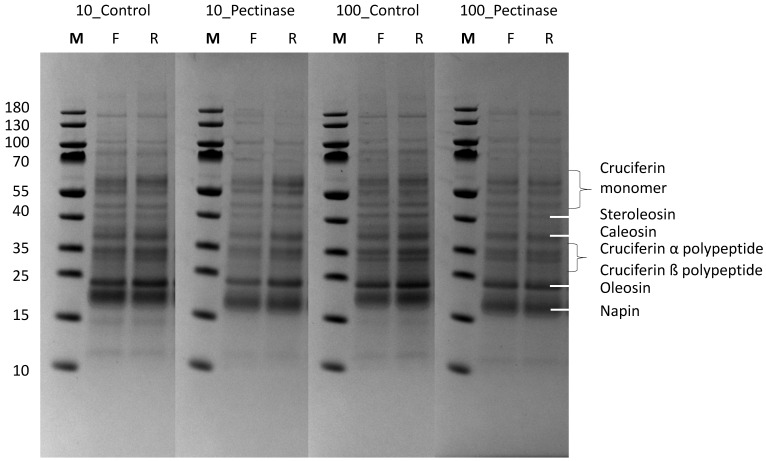
SDS-PAGE of rapeseed protein concentrates without and with pectinase after UF filtration with molecular cut-offs of 10 and 100 kDa. The gel was run under non-reducing conditions, and F and R indicate feed and retentate, respectively. M is molecular marker. Loaded with equal protein concentrations.

To further investigate the effect of ultrafiltration on the protein profile, the rapeseed protein concentrates and their corresponding retentates after filtration were subjected to gel electrophoresis, as depicted in Figure 2. The gels were loaded with equal amounts of protein and ran under non-reducing conditions to best highlight the differences in their polypeptide profile. Figure 2 illustrates the presence of the rapeseed storage proteins, including cruciferins (20–60 kDa) and napins (14–18 kDa), and the oleosomes-associated oleosins (20 kDa) [26,27] in all collected fractions. In addition, bands with molecular weights >70 kDa were found in all samples, highlighting protein aggregation. Comparing the feed and the corresponding retentate collected at the end of filtration reveals higher band intensities in the post-filtration samples, suggesting protein concentration as a result of ultrafiltration. There was no detectable protein in the permeate. These findings contrast with prior research conducted by Ntone et al. (2021), who isolated napin (14 kDa) from rapeseed using a 100 kDa UF membrane. This difference could be attributed to the lower pH used in our study, potentially causing protein aggregation or protein polysaccharide complexation. To investigate whether the absence of napin permeation was due to fouling-induced changes in membrane selectivity or alterations in the colloidal structure of napin resulting from extraction conditions, an additional filtration experiment was conducted. In this test, a 100 kDa ultrafiltration (UF) membrane was utilized on a vibrating filtration unit. The application of vibrations helps prevent material deposition on the membrane, reducing fouling. Despite using this setup, no permeation of napin was observed, suggesting that the colloidal structure of napin restricts its passage through the 100 kDa pores. 

### 3.2. Filtration Performance 

**Figure 3:**
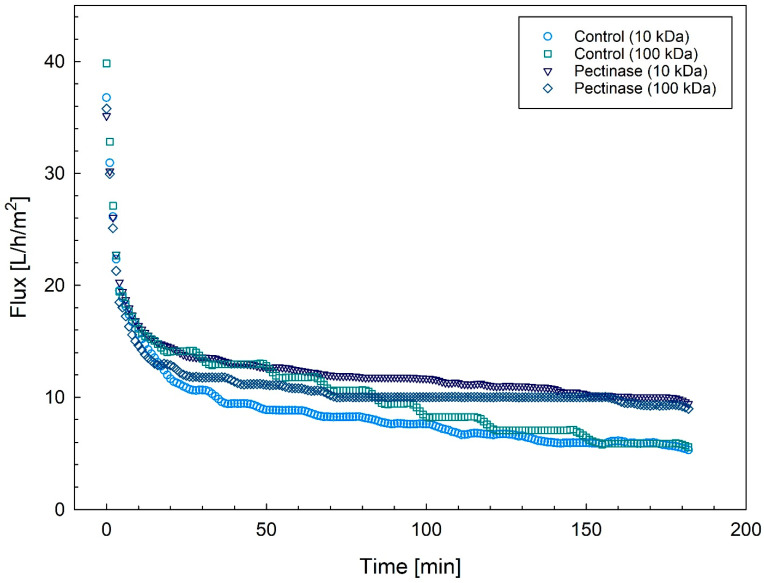
Effect of pectinase treatment on the permeate flux measured during the filtration of rapeseed protein concentrates without (circle and squares) and with pectinase (triangle and rhombus) using 10 kDa (circle and triangle) and 100 kDa (squares and rhombus) UF membranes. The curves represent the average of two independent replicates.

To evaluate changes in the membrane resistance, permeate fluxes were measured during filtration, and the changes occurring over time are presented in Figure 3. The fluxes showed a decline as a function of time, which could be distinguished in three defined stages. Following an initial rapid decline in permeate fluxes lasting approximately 6 min at the onset of filtration (stage I), a subsequent gradual decrease was noted throughout the rest of the filtration process. This decline can be categorized into two different stages: stage II spanning from 6 to 140 min, and stage III lasting the remaining 40 min. The decline in permeate flux for the two initial stages, described as the change in permeate flux per minute, is summarized in Table 2. The findings revealed a comparable reduction in permeate fluxes during the initial phase (stage I) with no effect of pectinase. This occurred with both UF membranes, with tight (10 kDa) and open (100 kDa) pores, resulting in flux reductions of 3 J/min and 5 J/min, respectively. The decrease in permeate flux was less pronounced during filtration with smaller-sized pores. In the intermediate stage of filtration, stage II, the flux decline was not affected by the membrane pore size. This second stage of filtration is linked to the formation of a cake layer, which introduces an additional layer of resistance and decreased the membrane selectivity [11]. The decrease in permeate flux in this stage was about 0.09 J/min. When pectinase was added to the concentrate, the permeate flux was less affected, with a decline of about 0.06 J/min, regardless of pore size. Interestingly, feed streams with the same compositional properties, irrespective of pore size, reached the same flux level after 180 min (Figure 3). However, there was a notably higher end flux for extracts carried out in the presence of pectinase. Based on these findings, it was concluded that carbohydrates played a key role in the formation of the cake layer, which in turn clearly limited the membrane selectivity. 

### 3.3. Fouling Behavior 

To assess fouling resistance, the parameters of total fouling, reversible fouling, irreversible fouling, and flux recovery were calculated. The results are shown in Table 2. The addition of commercial pectinase during extraction produced a feed, which showed a reduced overall fouling compared to the non-pectinase-treated counterpart. Table 2 shows that the fouling observed in both types of membranes (10 and 100 kDa UF membranes) formed by the control rapeseed protein concentrate was mainly irreversible, exceeding 68%. On the other hand, the pectinase-treated counterpart filtered through the 10 kDa UF membrane displayed a significant reduction in irreversible fouling, which was approximately 40%. This observation suggests that the incorporation of pectinase during extraction induced significant compositional and structural changes, enhancing the filtration performance, and that such treatment shows great potential for controlling membrane filtration performance. However, even for the pectinase-treated extracts, there was still a substantial reversible fouling effect. The high level of reversible fouling was also reflected in the flux recovery values (Table 2), which were higher for the pectinase extract compared to the control rapeseed concentrate. Only about 30% of the flux could be recovered with chemical cleaning, regardless of pore size, apart from the pectinase-treated process, where approximately 60% of the flux was recovered when filtration was conducted with the 10 kDa UF membrane.

### 3.4. Chemical Characterization of Irreversible Foulants

**Figure 4:**
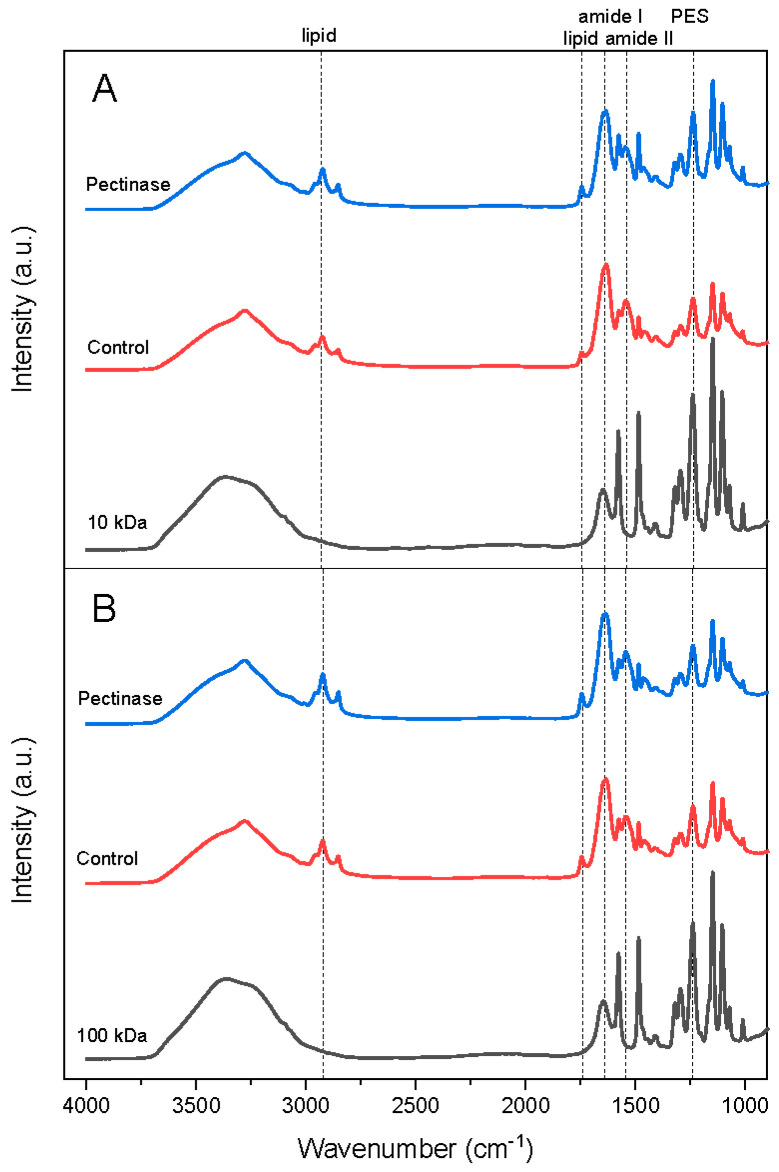
ATR-FT-IR spectra of new and fouled PES UF membrane with a molecular cut-off at (**A**) 10 kDa and (**B**) 100 kDa. Membranes after filtration of control rapeseed protein concentrates and concentrates treated with pectinase. Bottom spectra are new membranes. The spectra are the average of two replicates measured at three locations on the membrane.

ATR-FT-IR analysis was utilized to identify the main components contributing to the irreversible fouling of the membranes. Spectra were recorded for both new and fouled membranes following the cleaning procedure. Multivariate analysis on the spectra clearly demonstrated that there was a significant difference between the new and fouled membranes. This was evident by the distinct clustering of samples during PCA. Differences in the chemical spectra between new and fouled membranes are shown in Figure 4 with averaged spectra. The native PES membranes exhibited multiple peaks across the entire spectrum, assigned to the chemical constituents, as previously published [14,28]. A characteristic peak (highlighted at 1240 cm^−1^) was attributed to the asymmetric stretch of the aromatic ether in the PES structure [14,28]. This peak was also detected in the fouled membranes but with reduced intensities due to shadowing from fouled components. Two peaks (1720–1770 cm^−1^ and 2890–2980 cm^−1^), indicative of lipid presence [14], were exclusively found in the fouled membranes. These lipid-associated peaks appeared in all used membranes, irrespective of pore size or the addition of pectinase during filtration, clearly demonstrating that lipid molecules play an important role in the fouling of these membranes even though the rapeseed concentrates had a low lipid content [21]. Furthermore, an amide I (1600–1700 cm^−1^) and amide II peak (center at 1540 cm^−1^) indicated the presence of proteins in all fouled membranes. However, the amide I peak was also present in the native membranes due to the benzene ring stretching within the PES structure [14,28]. It was expected that proteins, due to their small globular structure and size, and especially napin, would permeate through the membranes, and if existing as colloidal aggregates, may also contribute to the formation of a fouling layer. Mass balances showed that rapeseed proteins were retained on the retentate side, and gel electrophoresis further confirmed the absence of protein in the permeates. The lack of protein concentration in the retentate suggested that protein was lost during the process, likely due to its entrapment within the membrane pores, irrespective of the size of the UF membrane. 

To explore potential variations in the protein composition among the different fouling layers, deconvolution of the amide I band was performed to highlight potential differences in their secondary structures. Results from the deconvolution data are reported in Table 3. The high standard deviations indicated substantial variation in the secondary structural components of proteins within the fouling layer, as FTIR measurements were conducted at various spots on the surface of each membrane. The identified bands corresponding to intermolecular (peak center at 1611–1625 cm^−1^, 1670–1682 cm^−1^ and 1690 cm^−1^) and intramolecular (peak center at 1623–1642 cm^−1^ and 1680 cm^−1^) β-sheets, random coils (1633–1661 cm^−1^), α-helices (1644 to 1660 cm^−1^) and β-turns (1654 to 1684 cm^−1^) were present in the majority of the fouling layers. These peak assignments align with established literature values [29]. There were no significant differences between the treatments, regardless of membrane pore size (10 and 100 kDa) or feed stream (with or without pectinase), except from random coil structures which were more pronounced in the fouling layers in the tight UF membranes. It is important to point out that the cleaning process, which exposed the membranes to high alkaline and acidic solutions, may change the structure of the residual proteins present in the irreversible fouling [30]. Furthermore, ATR-FT-IR analysis was performed on the membrane surface and is therefore biased to components adhering to the outer surface of the membrane. The chemical analysis of the membrane layer indicated no differences in the composition of the material deposited regardless of the use of pectic polysaccharides. In all cases, the membrane fouling layer consisted of lipids and protein. However, as shown in Table 2, the addition of pectinase in the extraction resulted in a lower extent of fouling. It may therefore be hypothesized that the impact of pectinase extends beyond modifying the colloidal structure of pectic polysaccharides, potentially influencing the colloidal assembly of rapeseed proteins. Rapeseed proteins, and especially napin, are known to form complexes with pectins [31], and previous studies have reported that pectin hydrolysis promotes protein complexation [21].

The findings of this study suggest that the combined application of pectinase and membrane filtration can serve as an effective strategy to limit fouling and enhance process performance. However, further research is required to understand the interactions between proteins and the membrane, aiming to mitigate protein-induced fouling. 

## 4. Conclusions 

The present work shows the potential of ultrafiltration as a means to further concentrate a mildly processed rapeseed protein concentrate obtained from an aqueous extraction. Regardless of the pore size, the PES membranes retained all proteins while allowing the passage of small molecular weight molecules, such as carbohydrates. There was a significant decrease in the solids content of the retentate after filtration. The use of larger pore sizes led to a higher extent of fouling, as evidenced by a reduction in permeate flux, with increasing levels of irreversible fouling. The addition of a commercial pectinase during extraction improved the filtration performance notably and reduced irreversible fouling significantly, especially for the 10 kDa PES membrane. These findings suggest that co-extracted carbohydrates play a crucial role in creating a cake layer during filtration, significantly reducing permeate fluxes. Chemical analysis using FTIR on the used membranes after cleaning highlighted the presence of lipids and proteins in the irreversible fouling layer, regardless of whether pectinase treatment was applied. 

This study, albeit only carried out at a small laboratory scale, served as a means to compare the effect of pectinase during extraction, and it demonstrated how the use of enzyme-assisted extraction of the protein concentrate results in changes in the filtration performance. Although filtration serves as a gentle method for fractionating and concentrating protein in feed streams, these results emphasize the importance of understanding the compositional and structural peculiarities of the feed to tailor the filtration process effectively. More work is needed to better understand how to optimize the utilization of raw plant-based extracts as feed streams in these processes. Furthermore, this study highlights the need for a deeper understanding of the colloidal protein structures that contribute to irreversible fouling and their specific interactions with the membrane.

## Figures and Tables

**Table 1:** Effect of pectinase treatment on the composition of retentates after ultrafiltration using a 10 and 100 kDa membrane. The concentration factor is based on permeate volume (Equation (1)). Concentration ratios are defined as the concentration in the final retentate divided by the concentration in the initial feed. Dry matter recovery in the permeate is reported as the percentage recovered in the permeate relative to the initial dry matter in the feed. Data are the average of two replicates ± standard deviation.

	Feed			Concentration Factor		Retentate				Permeate	
	Dry Matter (g/100 g)	Protein (g/100 g dm)	Carbohydrates (g/100 g dm)			Dry Matter Conc. Ratio		Carbohydrates Conc. Ratio		Dry Matter Recovery (%)	
				10 kDa	100 kDa	10 kDa	100 kDa	10 kDa	100 kDa	10 kDa	100 kDa
Control	1.6 ± 0.1	37.0 ± 1.3	52 ± 5	1.23 ± 0.01	1.33 ± 0.01	0.95 ± 0.01	1.04 ± 0.00	0.98 ± 0.04	0.84 ± 0.01	2.5 ± 1.5	7.9 ± 0.6
Pectinase	1.4 ± 0.1	33.7 ± 1.3	60 ± 11	1.43 ± 0.02	1.39 ± 0.02	1.04 ± 0.03	0.98 ± 0.01	0.76 ± 0.08	0.73 ± 0.06	7.0 ± 2.5	9.3 ± 1.5

**Table 2:** Effect of pectinase treatment on the decrease in permeate flux and fouling behavior reported as percentage change in flux after ultrafiltration using 10 and 100 kDa membranes. Stage I defines the first 6 min of filtration, and stage II is from 6 to 140 min. Data are the average of two replicates ± standard deviation.

Feed Stream	MWCO (kDa)	Flux Decrease (L/h/m^2^/min)		Total Fouling (%)	Reversible Fouling (%)	Irreversible Fouling (%)	Flux Recovery (%)
		Stage I	Stage II				
Control	10	3.93 ± 0.20	0.09 ± 0.00	96.9 ± 1.3	28.4 ± 13.8	68.5 ± 15.2	31.5 ± 15.2
	100	5.9	0.09	97.9 ± 0.2	24.4 ± 1.9	73.5 ± 2.2	26.5 ± 2.2
Pectinase	10	3.15 ± 0.39	0.06 ± 0.00	91.9 ± 0.6	51.7 ± 2.1	40.2 ± 1.5	59.8 ± 1.5
	100	5.51 ± 0.59	0.05 ± 0.00	95.8 ± 0.1	27.8 ± 3.2	68.0 ±3.0	32.0 ± 3.0

**Table 3:** Deconvolution of the amide I region and the corresponding secondary structures. Values are given as the area of the deconvoluted peak (%) and are the average of two replicates measured at three locations on the membrane ± standard deviation. Letters indicate significant variations (*p* < 0.05).

Feed Stream	MWCO (kDa)	β-Sheet, Intermolecular	β-Sheet, Intramolecular	Random Coil	α-Helix	β-Turn	β-Sheet, Intermolecular	β-Sheet, Intramolecular	β-Sheet, Intermolecular
Control	10	7.74 ± 3.42 ^a^	32.40 ± 7.86 ^a^	1.19 ± 1.26 ^a^	30.23 ± 24.90 ^a^	22.51 ± 18.51 ^a^	3.48 ± 2.06 ^a^	6.57 ± 2.19 ^a^	7.74 ± 3.42 ^a^
	100	14.26 ± 8.66 ^a^	29.56 ± 7.56 ^a^	39.78 ± 23.00 ^b^	12.74 ± 21.96 ^a^	16.24 ± 15.06 ^a^	3.79 ± 2.42 ^a^	-	14.26 ± 8.66 ^a^
Pectinase	10	13.97 ± 6.34 ^a^	30.88 ± 15.73 ^a^	4.43 ± 6.92 ^a^	12.09 ± 18.62 ^a^	33.04 ± 18.27 ^a^	4.74 ± 1.33 ^a^	-	13.97 ± 6.34 ^a^
	100	14.31 ± 6.04 ^a^	21.89 ± 12.84 ^a^	31.94 ± 27.17 ^ab^	32.12 ± 26.82 ^a^	24.12 ± 22.00 ^a^	4.81 ± 1.16 ^a^	-	14.31 ± 6.04 ^a^

## Data Availability

The original contributions presented in the study are included in the article, further inquiries can be directed to the corresponding authors.

## Abbreviations

CIP	Cleaning-in-place
CP	Concentration polarization
IEP	Isoelectric precipitation
MF	Microfiltration
MWCO	Molecular weight cut-off
PES	Polyethersulfone
PS	Polysulfone
PCA	Principal component analysis
TMP	Transmembrane pressure
UF	Ultrafiltration
